# Cross-site validation of lung cancer diagnosis by electronic nose with deep learning: a multicenter prospective study

**DOI:** 10.1186/s12931-024-02840-z

**Published:** 2024-05-10

**Authors:** Meng-Rui Lee, Mu-Hsiang Kao, Ya-Chu Hsieh, Min Sun, Kea-Tiong Tang, Jann-Yuan Wang, Chao-Chi Ho, Jin-Yuan Shih, Chong-Jen Yu

**Affiliations:** 1 Department of Internal Medicine, National Taiwan University Hospital, Taipei, Taiwan; 2 Department of Internal Medicine, National Taiwan University Hospital Hsin-Chu Branch, Hsin-Chu, Taiwan; 3 Department. of Electrical Engineering, National Tsing Hua University, No. 101, Sec. 2, Kuang-Fu Road, Hsinchu, 30013 Taiwan

**Keywords:** Electronic nose, Cross-site validation, Lung cancer, Breathprint, Deep learning, Data augmentation

## Abstract

**Background:**

Although electronic nose (eNose) has been intensively investigated for diagnosing lung cancer, cross-site validation remains a major obstacle to be overcome and no studies have yet been performed.

**Methods:**

Patients with lung cancer, as well as healthy control and diseased control groups, were prospectively recruited from two referral centers between 2019 and 2022. Deep learning models for detecting lung cancer with eNose breathprint were developed using training cohort from one site and then tested on cohort from the other site. Semi-Supervised Domain-Generalized (Semi-DG) Augmentation (SDA) and Noise-Shift Augmentation (NSA) methods with or without fine-tuning was applied to improve performance.

**Results:**

In this study, 231 participants were enrolled, comprising a training/validation cohort of 168 individuals (90 with lung cancer, 16 healthy controls, and 62 diseased controls) and a test cohort of 63 individuals (28 with lung cancer, 10 healthy controls, and 25 diseased controls). The model has satisfactory results in the validation cohort from the same hospital while directly applying the trained model to the test cohort yielded suboptimal results (AUC, 0.61, 95% CI: 0.47─0.76). The performance improved after applying data augmentation methods in the training cohort (SDA, AUC: 0.89 [0.81─0.97]; NSA, AUC:0.90 [0.89─1.00]). Additionally, after applying fine-tuning methods, the performance further improved (SDA plus fine-tuning, AUC:0.95 [0.89─1.00]; NSA plus fine-tuning, AUC:0.95 [0.90─1.00]).

**Conclusion:**

Our study revealed that deep learning models developed for eNose breathprint can achieve cross-site validation with data augmentation and fine-tuning. Accordingly, eNose breathprints emerge as a convenient, non-invasive, and potentially generalizable solution for lung cancer detection.

**Clinical trial registration:**

This study is not a clinical trial and was therefore not registered.

**Supplementary Information:**

The online version contains supplementary material available at 10.1186/s12931-024-02840-z.

## Introduction

Lung cancer remains a predominant cause of cancer-related mortality worldwide, accounting for an estimated 2.2 million new cases and 1.8 million deaths in 2020 [1]. In its early stages, lung cancer often presents no symptoms, making it challenging to detect during routine health examinations. Although low-dose computed tomography (CT) of chest has been employed for lung cancer screening to facilitate earlier diagnosis and reduce mortality, a significant number of lung cancer patients remain undiagnosed until the disease has advance [2]. Furthermore, low-dose CT of chest has its limitations, including high cost, radiation exposure, and limited availability in many clinics. Consequently, there is a pressing need for a non-invasive, cost-effective, and readily accessible screening tool for early detection of lung cancer.

Electronic nose (eNose) is a novel device using sensors to generate breathprints that reflect patterns of volatile organic compounds [3]. eNose has the advantage of being non-invasive, easy to operate, short turnaround time and point-of-care. eNose has been applied in diagnosis of various diseases, encompassing communicable diseases such as COVID-19, tuberculosis and non-communicable diseases including diabetes and cancer. eNose has also been investigated in lung cancer diagnosis and treatment monitoring in previous studies.

Earlier studies evaluating eNose in lung cancer detection were mainly single center and compare between lung cancer and healthy control [4]. Previous studies also have shortcomings of lack of validation, especially cross-site validation [5]. While breathomics are prone to change in environment, external validation remains a major obstacle to clinical application. While more recent studies usually include a multicenter design of recruiting participants, cross site and independent validation were still not readily available [6, 7].

On the other hand, algorithms for eNose breathprint analysis is also in evolution [8]. Deep learning involving convoluted neural network (CNN) is novel and emerging technique for breathprint analysis [8, 9]. Some analytic approaches such as transfer learning and data augmentation have been applied in other aspects of biomedical imaging researches [10]. These methods could potentially propagate sample size, enhance performance and ameliorate the drop of performance in domain shift [11, 12]. Most eNose studies have not yet incorporated this into analytic methods of eNose breathprint for lung cancer identification.

This study, therefore, aimed to validate eNose breathprint for lung cancer diagnosis in a cross-site setting with deep learning techniques including data augmentation and fine-tuning incorporated into the analytic methods. We aimed to expand generalizability of eNose breathprint in lung cancer diagnosis and advance eNose further in clinical practice.

## Methods

### Patient selection and study setting

This study was conducted prospectively at two facilities: the National Taiwan University Hospital (NTUH; test cohort, S2, site 2) and its Hsin-Chu branch (NTUH-HC; training/validation cohort, S1, site 1), both of which are referral centers for individuals with lung cancer and lung cancer suspects in Taiwan. The NTUH, a 2300-bed medical center in northern Taiwan, and the NTUH-HC, a regional hospital located 60 km away with a 700-bed capacity, have actively participated in eNose breathprint studies. The personnel at these institutions are well-acquainted with the eNose collection process and equipment operation. The institutional review boards (IRB) of participating hospitals approved this study (IRB no. 202112057RINB, 108-011-E). Inform consent was obtained from all participants who agreed to participate in this study.

For this study, we enlisted participants from three groups: individuals diagnosed with lung cancer, healthy controls, and diseased controls with either structural lung diseases confirmed on chest CTs or spirometry-confirmed chronic obstructive pulmonary disease. We confirmed the absence of lung cancer in the diseased control group through chest CT imaging and follow-up evaluations. During a two-year follow-up period, all control participants, encompassing both healthy and diseased controls, remained free from lung cancer.

### Definition of diseases and data collection

For lung cancer patients, pathological confirmation was required for establishing the diagnosis. The stage was classified according to the 8th edition of the American Joint Committee on Cancer staging system for lung cancer [13]. We collected the data from a prospectively maintained database and medical records. Comorbidities included chronic obstructive pulmonary disease (COPD), asthma, diabetes mellitus (DM), and end-stage renal disease (ESRD). For healthy participants, a screening interview was performed to exclude underlying lung diseases and smoking habits. Chest x-rays of healthy participants, if available, were also reviewed to exclude structural lung disease. For diseased controls, participants must have either structural lung diseases confirmed on chest computed tomography or spirometry-confirmed chronic obstructive pulmonary disease.

### Breath sample collection

The breath sample collection process has been described in our previous study [9]. Briefly, the breath sampling system included a one-way VBMax™ filter and two one-litre multi-layer foil gas sampling bags. Participants fasted for 4 h and avoided smoking and alcohol before testing. Each individual took a deep breath, then used the blow-to-breath sampling system connected to two Robert Clamps: the first collecting dead space air (not analyzed) and the second collecting end-tidal breath for analysis.

### Breath analysis using eNose

The eNose system, developed by SEXTANT (Enosim Bio-Tech Co., Ltd., Hsinchu City, Taiwan), builds upon previous work and incorporates a total of 14 metal-oxide gas sensors. This system, which also includes flow meters and temperature and humidity sensors, is designed to work seamlessly with the necessary interface circuits. Leveraging Metal-Oxide-Semiconductor (MOS) gas sensors sourced from Figaro USA, Inc. and Nissha FIS, Inc., the SEXTANT system operates based on oxidation-reduction sensing mechanisms. These sensors have been enhanced with different materials to optimize both selectivity and sensitivity in detecting various gases [9]. A video describing the process of breath analysis using eNose is also available as Additional File 1: Supplementary Video.

### CNN model construction

For eNose breathprint, we first pre-processed the raw data of eNose into 14-channel $16\times 16$ images and use a parallelizable calculation model, the convolution neural network, as the training model. We chose the rectified linear units (ReLUs) as the activation function to improve the training speed, and applied three layers of CNN to extract binary output from input images. Positive and negative outputs refer to whether this patient has lung cancer or not, respectively. The structure of CNN is shown in Additional File 2: Figure S1

### Data augmentation and fine-tuning

In this study, we applied two methods of data augmentation including Semi-supervised Domain Generalized (Semi-DG) Augmentation (SDA) and Noise-Shift Augmentation (NSA) methods. In SDA, Fourier transformation was applied and while in the NSA, we added Gaussian noise to the breathprint and performed a backward shift operation [14–17]. The detailed techniques of data augmentation were described in Additional File 3: Supplementary File, Additional File 4: Figure S2 and Additional File 5: Figure S3. We augmented eNose breathprint at an 1:1 ratio.

For fine-tuning, we first trained the model on the training cohort to obtain the initial weight of the model. Then, we used 10 test cohort to fine-tune the model to obtain new model weights. We chose to fine-tune our dataset using 10 samples based on our previous study, where we aimed to use a small proportion of our dataset, approximately 10–20% of the samples, for tuning [18]. We also conducted another analysis using 20 samples but observed only marginal improvement in the results. Additionally, data used for fine-tuning were separated from the test data and not used for testing.

### Dataset definition and analytic flow

**Fig. 1:**
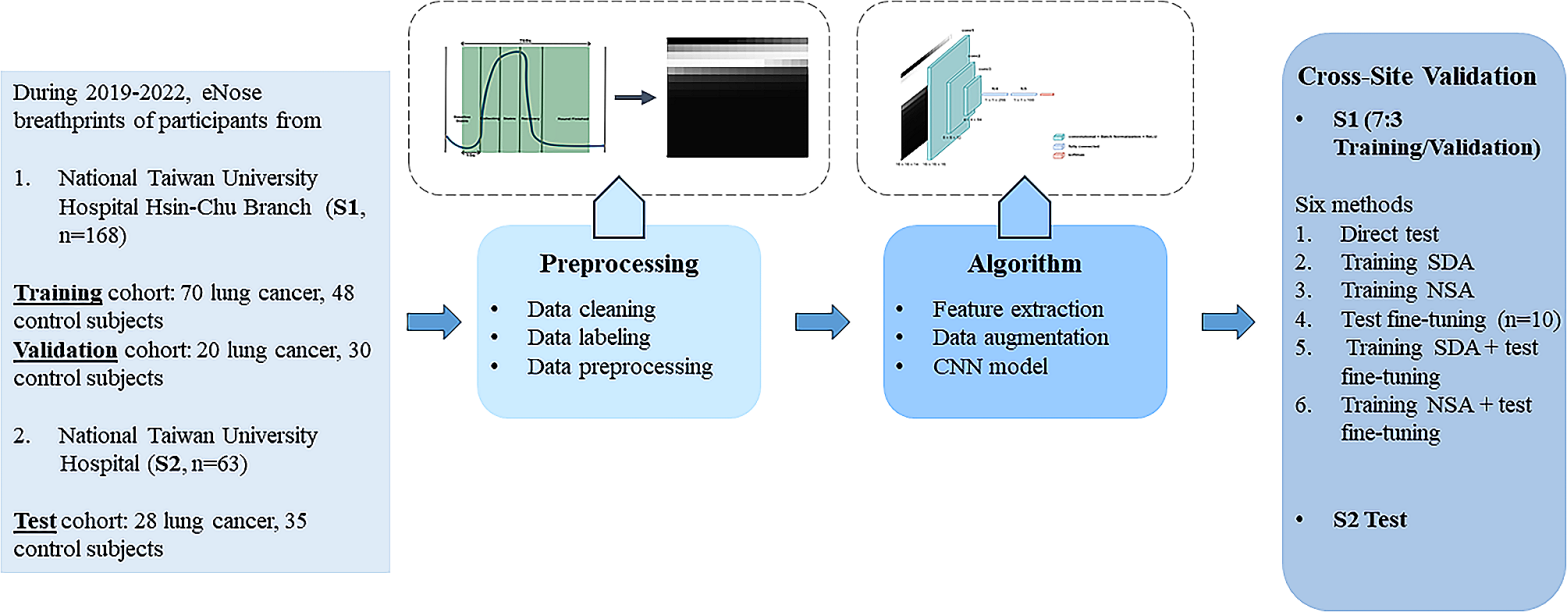
Flowchart and analytic flow. CNN, convoluted neural network; NSA, noise-shift augmentation; SDA, semi-supervised domain generalized augmentation

The training cohort was divided at 7:3 ratio, with 70% used for model training and the remaining 30% for model validation, according to time frame of recruitment. For the analysis, data augmentation (at a 1:1 ratio) was applied to the training portion. After training and validation, the model was tested with or without fine-tuning on the test dataset. The rest of the test dataset served to evaluate the model’s performance. The detailed process was described in Fig. 1.

## Statistical analysis

All variables were presented as either numbers (percentages) or as the mean ± standard deviation, depending on their nature. For categorical variables, the chi-square test was employed. For continuous variables, either the student’s t-test or the one-way analysis of variance (ANOVA) was used for comparison. To evaluate the model’s performance, we assessed accuracy, sensitivity, and specificity. Additionally, the area under the receiver operating characteristic (AU-ROC) curves were constructed to showcase the model’s performance. Confidence intervals (CI) were provided for analysis using the bootstrapping procedure. For the machine learning method, we used the scikit-learn package (version 0.23.2) in Python (version 3.8.5). All p-values were two-sided, with statistical significance set at *p* < 0.05.

## Results

### Demographics of participants

A total of 231 participants were enrolled (168 in the training/validation cohort (Site 1, National Taiwan University Hospital Hsin-Chu branch cohort) and 63 in the test cohort (Site 2, National Taiwan University Hospital cohort)). Table 1. describes the demographic data of all participants in the training, validation and test cohort. In the training cohort (S1), there were 70 (59.3%) lung cancer patients and 48 (33.9%) non-lung cancer control subjects (including 10 healthy control and 38 diseased control). In the validation cohort (S1), there were 20 (40%) lung cancer and 30 (60%) control subjects (including 6 healthy control and 24 diseased control). On the other hand, there were 28 (44.4%) lung cancer and 35 (55.6%) non lung cancer patients (including 10 healthy control and 25 diseased control) in the test cohort (S2).

**Table 1:** Clinical characteristics of participants

	All	Training Cohort (S1, site 1, Hsin-Chu cohort)				Validation Cohort (S1, site 1, Hsin-Chu cohort)				Test Cohort (S2, site 2, Taipei cohort)				Training vs. Validation cohort	Training vs. Test Cohort
	All (*n* = 231)	All (*n* = 118)	Lung cancer (*n* = 70)	Control Subjects (*n* = 48)	*P* value	All (*n* = 50)	Lung cancer (*n* = 20)	Control Subjects (*n* = 30)	*P* value	All (*n* = 63)	Lung cancer (*n* = 28)	Control Subjects (*n* = 35)	*P* value	P* value	P** value
Sex (Male/Female)	117 (50.7)/ 114 (49.4)	58 (49.2)/60 (50.8)	39 (55.7)/31 (44.3)	19 (39.6)/29 (60.4)	0.0851	26 (52)/24 (48)	8 (40)/12 (60)	18 (60)/12 (40)	0.1655	33 (52.4)/ 30 (47.6)	18 (64.3) / 10 (35.7)	15 (42.9)/ 20 (57.1)	0.0906	0.7357	0.8968
Age (mean ± SD)	61.3 ± 15.5	61.0 ± 15.2	63.4 ± 11.9	57.6 ± 18.6	0.0615	62.9 ± 17.1	66.9 ± 12.7	60.3 ± 19.3	0.1859	60.4 ± 14.7	63.1 ± 12.6	58.2 ± 16.1	0.1908	0.4823	0.6742
Smoking					0.0095				0.2699				0.3024	0.8841	0.4478
Current smoker	33 (14.3)	15 (12.7)	7 (10)	8 (16.7)		5 (10)	1 (5)	4 (13.3)		13 (20.6)	7 (25.0)	6 (17.1)			
Ex-smoker	47 (20.4)	23 (19.5)	20 (28.6)	3 (6.3)		10 (20)	6 (30)	4 (13.3)		14 (22.2)	8 (28.6)	6 (17.1)			
Never smokers	151 (65.4)	80 (67.8)	43 (61.4)	37 (77.1)		35 (70)	13 (65)	22 (73.3)		36 (57.1)	13 (46.4)	23 (65.7)			
DM	30 (13.0)	13 (11.0)	8 (11.4)	5 (10.4)	0.8631	7 (14.0)	3 (15)	4 (13.3)	0.8679	10 (15.9)	6 (21.4)	4 (11.4)	0.2805	0.5851	0.6329
HTN	61 (26.4)	37 (31.4)	24 (34.3)	13 (27.1)	0.4074	14 (28)	7 (35)	7 (23.3)	0.3681	10 (15.9)	5 (17.9)	5 (14.3)	0.6999	0.6654	0.0762
COPD	41 (17.8)	13 (11.0)	8 (11.4)	5 (10.4)	0.8631	12 (24)	2 (10)	10 (33.3)	0.0915	16 (25.4)	7 (25.0)	9 (25.7)	0.9484	0.0306	0.0232
Cancer	125 (54.1)	71 (60.2)	70 (100)	1 (2.1)	< 0.0001	20 (40)	20 (100)	0	< 0.0001	34 (54.0)	28 (100)	6 (17.1)	< 0.001	0.0164	0.0563

COPD, chronic obstructive pulmonary disease; DM, diabetes mellitus; ESRD, end-stage renal disease; HTN, hypertension; SD, standard deviation

* compare between training and validation cohort

** compare between test and training cohort

In the training cohort, the smoking status were different between the lung cancer and control subjects. In the validation test, the demographic data were similar between the lung cancer and control subjects. In the test cohort, there is a slight female preponderance not reaching statistical significance in the lung cancer subjects compared with the control subjects (Table 1).

For the lung cancer patients in training/validation cohort, 70 (77.8%) were adenocarcinoma, 12 (13.3%) were squamous cell carcinoma, 4 (4.4%) were small cell lung cancer while 4 (4.4%) were other histology type. In the test cohort, 15 (53.6%) were adenocarcinoma, 4 (14.3%) were squamous cell carcinoma, 4 (14.3%) were small cell lung cancer while 5 (17.9%) were other histology type. The distribution of histology type was different in the training/validation cohort and test cohort (*p* = 0.0165). For cancer stage, the two cohorts were not different (*p* = 0.5444) while the majority was stage IV cancer patients (Additional File 3: Table S1).

### eNose breathprints PCA

**Fig. 2:**
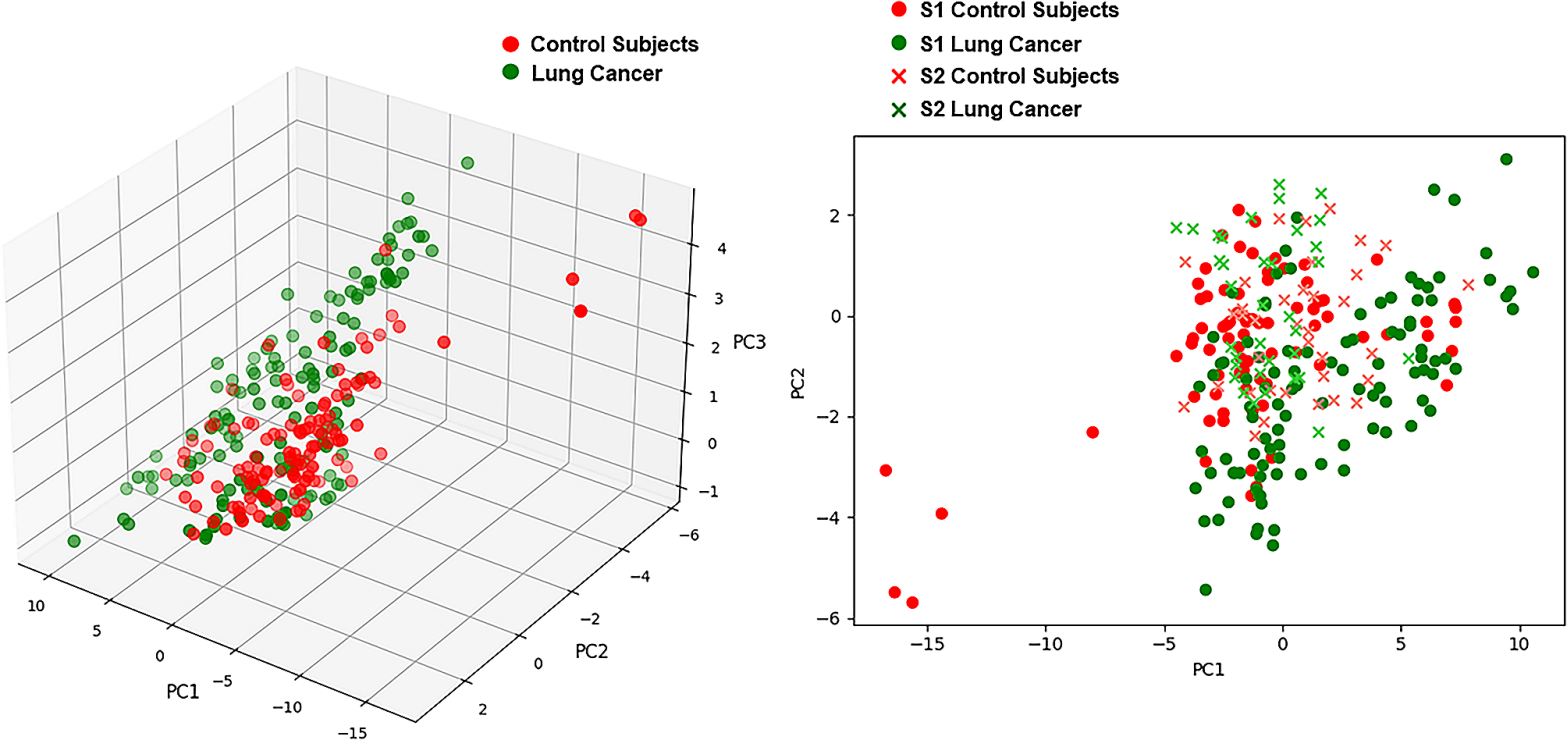
Principal component analysis plots of eNose breathprints

Figure 2 illustrates the PCA plots of breathprints in this study. Breathprints from the two individual sites were distinct. Within each site, the breathprints of both the lung cancer and non-lung cancer groups were interspersed and scattered.

### Performance of eNose

**Fig. 3:**
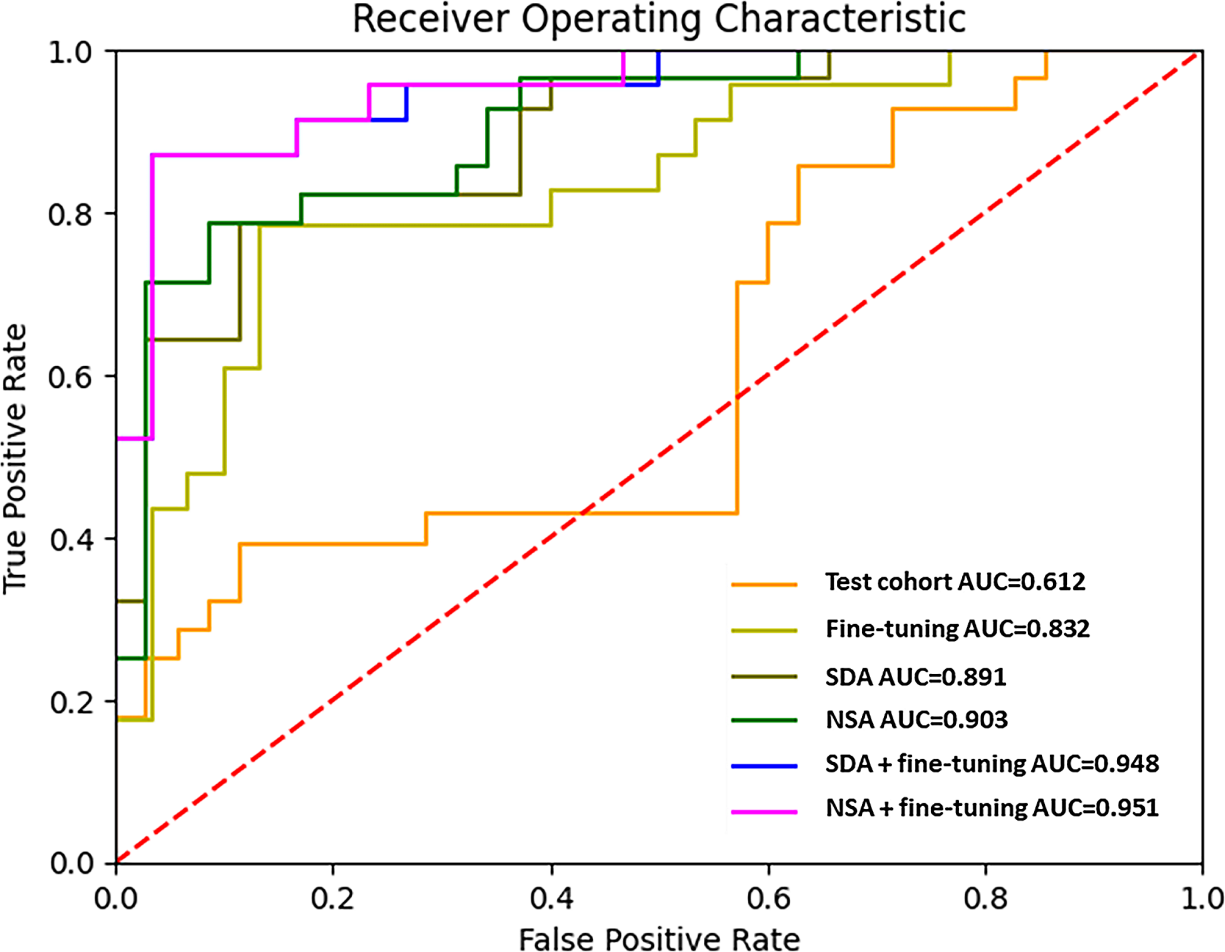
Area under the receiver operating characteristic curve of the test cohort (S2). AUC, area under the receiver operating characteristic curve; NSA, noise-shift augmentation; SDA, semi-supervised domain generalized augmentation

In the validation cohort (S1), the performance of eNose achieved an AUC of 0.89 (95% CI:0.84─0.93) with sensitivity of 0.90 (95% CI:0.85─0.95) and specificity of 0.83 (95% CI:0.73─0.87). While applying to the test cohort (S2), the performance was suboptimal with an AUC of 0.61 (95% CI:0.47─0.76), sensitivity of 0.43 (95% CI:0.36─0.50), specificity 0.43 (95% CI:0.37─0.54). With SDA, the AUC improved to 0.89 (95% CI: 0.81─0.97) with sensitivity of 0.82 (95% CI: 0.75─0.86) and specificity of 0.69 (95% CI: 0.60─0.80). With NSA, the AUC improved to 0.90 (95% CI: 0.83─0.98) with sensitivity of 0.82 (95% CI:0.75─0.86) and specificity of 0.69 (95% CI: 0.60─0.80). Applying fine-tuning, the AUC improved to 0.83 (95% CI: 0.72─0.94) and sensitivity of 0.78 (95% CI:0.70─0.83) and specificity of 0.6 (95% CI: 0.53─0.73). With SDA and fine-tuning, the performance further improved to AUC of 0.95 (95% CI: 0.89─1.00), sensitivity of 0.91 (95% CI: 0.83─0.96) and specificity of 0.77 (95% CI: 0.67─0.90). With NSA and fine-tuning, the performance also improved to AUC of 0.95 (95% CI: 0.90─1.00), sensitivity of 0.91 (95% CI: 0.83─0.96) and specificity of 0.77 (95% CI: 0.67─0.90) (Table 2). The AU-ROC of the test cohort (S2) is illustrated in Fig. 3.

**Table 2:** Diagnostic performance of eNose in the validation (S1) and test cohort (S2)

	AUC	95% CI	Sensitivity	95% CI	Specificity	95% CI	Accuracy	95% CI
Validation cohort (S1)	0.89	0.84─0.93	0.90	0.85─0.95	0.83	0.73─0.87	0.86	0.78─0.90
Test cohort (S2)	0.61	0.47─0.76	0.43	0.36─0.50	0.43	0.37─0.54	0.43	0.33─0.52
Test cohort (S2) with fine-tuning (*n* = 10)	0.83	0.72─0.94	0.78	0.70─0.83	0.60	0.53─0.73	0.68	0.60─0.75
Test cohort (S2) with SDA in training cohort (S1)	0.89	0.81─0.97	0.82	0.75─0.86	0.69	0.60─0.80	0.75	0.68─0.81
Test cohort (S2) with NSA in training cohort (S1)	0.90	0.83─0.98	0.82	0.75─0.86	0.69	0.60─0.80	0.75	0.68─0.81
Test cohort (S2) with SDA in training cohort (S1) and fine-tuning (*n* = 10) in test cohort (S2)	0.95	0.89─1.00	0.91	0.83─0.96	0.77	0.67─0.90	0.83	0.75─0.91
Test cohort (S2) with NSA in training cohort (S1) and fine-tuning (*n* = 10) in test cohort (S2)	0.95	0.90─1.00	0.91	0.83─0.96	0.77	0.67─0.90	0.83	0.75─0.91

NSA, noise-shift augmentation; SDA, semi-supervised domain generalized augmentation

Reversing the training/validation and test cohort (the training/validation cohort (S1) then became the test cohort, while the test cohort (S2) became the training validation cohort), we found that the performance of eNose achieved an AUC of 0.91 (95% CI: 0.81─1.00) with sensitivity of 0.89 (95% CI: 0.80─1.00) and specificity of 0.80 (95% CI: 0.60─1.00) in the new validation cohort. Again, the performance was unsatisfactory in the test cohort with an AUC of 0.56 (95% CI: 0.44─0.73), sensitivity of 0.63 (95% CI: 0.52─0.76), specificity 0.54 (95% CI: 0.48─0.60). SDA or NSA plus fine-tuning both achieved an AUC of 0.84 (95% CI: 0.78─0.90), sensitivity of 0.82 (95% CI: 0.73─0.90) and specificity of 0.79 (95% CI: 0.70─0.89) (Additional File 3: Table S2). The AU-ROC of the test cohort (S1) is illustrated in Additional File 6: Supplementary Fig. 4.

### Subgroup analysis

**Fig. 4:**
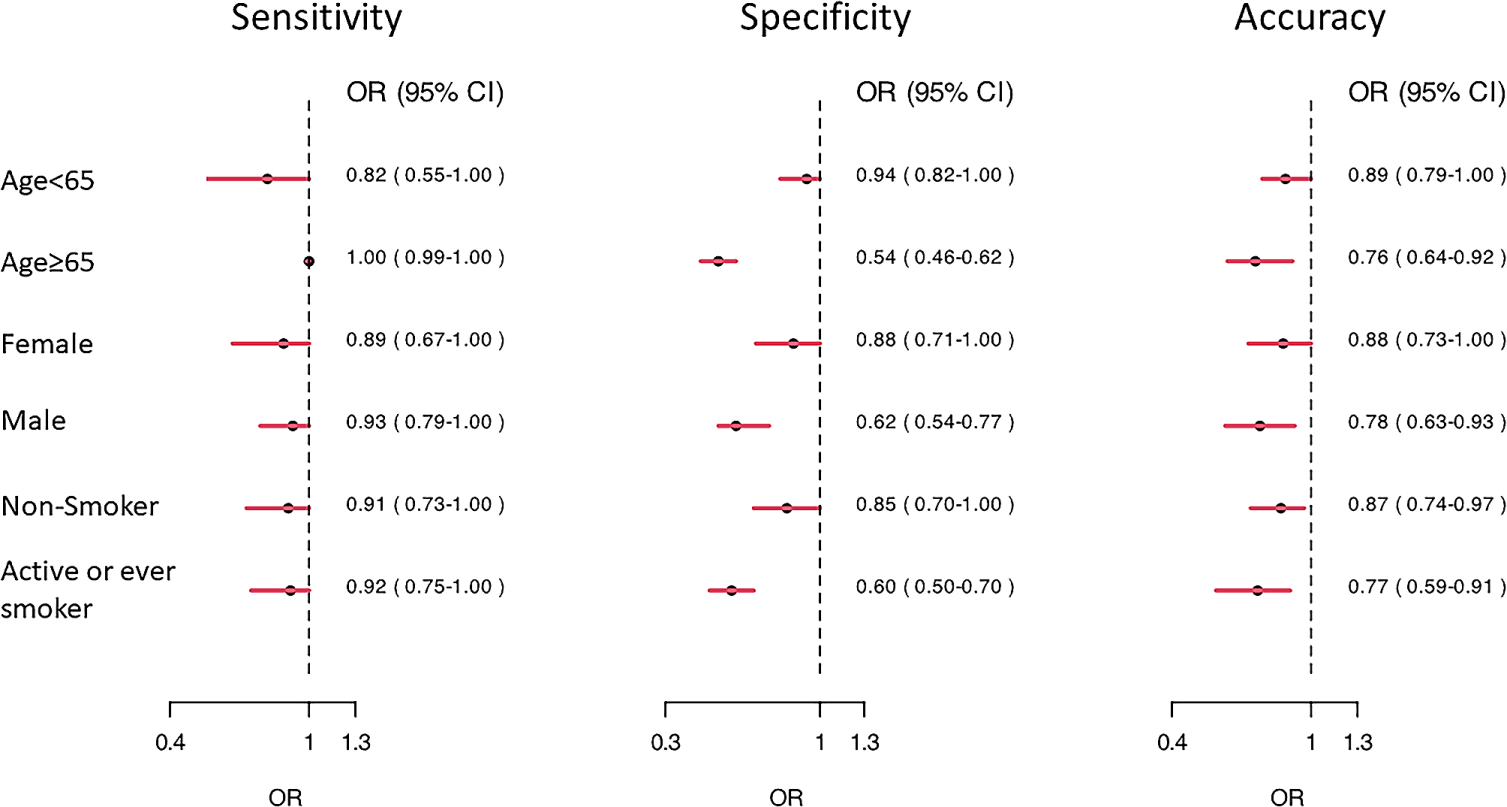
Forest plot of subgroup analysis. OR, odds ratio

In subgroup analysis (Fig. 4), we found that patients aged above 65-year-old had worse eNose performance compared with age less than 65-year-old (Accuracy: 0.76, 0.64─0.92 vs. 0.89, 0.79─1.00). While female and male patients had similar performance, the eNose had performed less satisfactory among those who ever or actively smoked than never smokers (Accuracy: 0.77, 95% CI: 0.59─0.91 vs. 0.87, 95% CI: 0.74─0.97). The performance was also best in the healthy control (accuracy: 1.00, 95% CI:0.89─1.00), followed by lung cancer patients (accuracy: 0.91, 95% CI:0.64─1.00) and diseased control patients (accuracy: 0.67, 95% CI:0.57─0.80). Among different histology types of lung cancer, the eNOSE correctly identifies all adenocarcinoma, SCLC, SqCC but incorrectly identifies two of the four lung cancer patients with other histologic classification.

Two patients were in their early stage (stage I and II) in the test cohort and they were all correctly classified as lung cancer (100%, 2/2). Also, the accuracy rate was 83.3% (5/6) for stage III lung cancer and 93.3% (14/15) for stage IV lung cancer in the test cohort.

Also, our model correctly identified 16 out of 17 (94.1%) lung cancer patients under treatment and 5 out of 6 (83.3%) fresh lung cancer patients not yet receiving anti-cancer treatment.

Detailed subgroup analysis of age, smoking status and comorbidities were further described in Additional File 3: Table S3.

## Discussion

In our study, we found that combining deep learning with transfer learning and data augmentation enables eNose to effectively tackle cross-site validation challenges. Using an eNose model trained at one site directly on another led to suboptimal results. Yet, by utilizing data augmentation and transfer learning, the eNose’s performance notably improved, achieving an AUC exceeding 0.9. As a result, electronic noses can accurately differentiate between lung cancer patients and those without the condition.

Breathomics has undergone extensive research for the purpose of detecting lung cancer. This approach is grounded in the theory that lung cancer patients may exhibit distinct metabolites and exhaled volatile organic compounds (VOCs) compared to persons without lung cancer [19]. In one prior investigation also conducted in the same participating hospital, the authors employed selected ion flow tube mass spectrometry (SIFT-MS) to identify and quantify 116 VOCs. Subsequently, the authors developed a predictive model for determining the likelihood of lung cancer based on quantitative VOC measurements. This approach yielded a commendable AUC and accuracy, with further enhancements achieved through the adjustment of confounding VOC effects [20]. It is worth noting, however, that this earlier study remained limited to a single-center setting and lacked external validation.

Cross-site validation of electronic nose has always been an important issue to be overcome. In earlier studies, the differentiation between lung cancer and non-lung cancer patients was performed without validation [4]. Some studies split one single cohort into training and validation part [5, 21, 22]. In one study, for instance, 199 participants were randomly split into an 80% training cohort and 20% validation cohort. A classification accuracy of 79% was subsequently attained by using XGBoost method [22]. In another study, by including 60 patients with lung cancer and 107 controls and assigning participants either to training or blinded validation cohort, the blinded validation cohort yielded diagnostic accuracy of 86%, sensitivity of 88% and specificity of 86%. For this approach, one may refer to the results of 86% accuracy obtained in our validation cohort.

Other studies used pooled data from multi-cohorts and then randomly split into training and validation cohort. In one study including multi-center cohorts with total of 575 patients, 376 patients were assigned to the training cohort and 199 patients assigned to the validation cohort. The training model then achieved an AUC-ROC of 0.79 (0.72–0.85) with a sensitivity of 88.2% and specificity of 48.3% in the validation. The study further achieved a better performance after integrating clinical data [6]. These approaches, however, do not really tackle with the issue of cross-site validation.

Cross-site validation is crucial due to several challenges associated with the generation of eNose breathprints. One significant challenge is the pervasive influence of environmental VOCs, which are constantly inhaled and participate in metabolic processes. This can modify the VOCs exhaled in human breath, subsequently affecting the generation of breathprints [20, 23]. Another challenge stems from the device itself, encompassing issues such as sensor drift and the complexities of achieving absolute calibration [24]. Although the PCA plot revealed a distinct breathprint distribution, it also highlighted the challenges of achieving cross-site validation. Our study indicated that using data-augmentation techniques could significantly reduce the load of data collection and improved model performance. With combination of fine-tuning using data from individual sites, the performance of eNose further improved. Importantly, in our research, we only utilized a small portion of the test dataset for fine-tuning, making a clinical approach feasible.

The appropriate selection of a control group is paramount in ensuring the validity of research findings. Differentiating between healthy individuals and those diagnosed with lung cancer may seem straightforward. However, such differentiation may not encapsulate the complexities of real-world scenarios. To enhance the representativeness of our study, we incorporated individuals with other pulmonary conditions into our control cohort. While smoking is predominantly identified as a primary risk factor for lung cancer among Caucasians, another distinct demographic—non-smoking Asian females with lung adenocarcinoma—emerges as notably susceptible [25]. In an effort to account for this, our control group integrated patients with structural lung disease primary consisting of bronchiectasis. Additionally, patients with COPD were incorporated into our cohort. By combining different groups with healthy people, we believe our control group more closely matches the variety of individuals with lung screenings in real life.

Subgroup analysis revealed that the eNose exhibited less satisfactory performance in elderly participants and smokers. This finding holds particular significance, as elderly participants often present with a higher prevalence of comorbidities compared to their younger counterparts. These comorbidities may have introduced complexity into the eNose breathprint profiles [26]. It is noteworthy that elderly patients constitute an emerging demographic among lung cancer patients, and early lung cancer detection could enhance the feasibility of surgical interventions and further improvement of performance of eNose may be warranted [27]. Additionally, it’s worth highlighting that eNose demonstrated less satisfactory performance in the smoker subgroup. This finding was consistent with our previous which also found inferior performance in the smoker group [9]. Considering smoking remains a major risk factor for lung cancer [28], Detecting lung cancer in individuals who smoke or have chronic obstructive pulmonary disease is crucial for early intervention and treatment of lung cancer [29]. Therefore, our findings highlight areas of weakness that need to be strengthened in our eNose device. eNose technology simulates the human olfactory system.

In real environments, gas mixtures can be influenced by numerous factors, such as environmental volatile organic compounds and humidity. Therefore, data enhancement methods are valuable as they can simulate these variations, making the model more adaptable and reducing the need for extensive data collection. Common data enhancement techniques for eNose encompass noise addition, data rotation and translation, and synthetic data generation. For instance, a study with focus on eNose’s classification of alternative herbal medicines employed several data enhancement strategies to minimize the heavy dependency on training materials [17]. One method involved augmenting the training dataset by adding Gaussian noise and data shifting [17]. In another study exploring the use of eNose to identify ripe tomatoes, the collected gas’s concentration value was converted into a grayscale value, synthesized into a grayscale image, and then augmented using methods such as cropping and zooming [30]. These data augmentation techniques successfully improved the performance of eNose.

There were studies utilizing data augmentation methods in human disease research to enhance domain generalization, bolster model robustness, and minimize overfitting risks. For instance, one study employed a continuous frequency domain spatial interpolation approach for data augmentation, achieving state-of-the-art results in retinal fundus and prostate magnetic resonance imaging segmentations [31]. More recently, another study explored six data augmentation techniques for electromyography signals: trial averaging, time slice recombination, frequency slice recombination, noise addition, cropping, and the use of a variational autoencoder. This research aimed to enrich data diversity, enabling the model to better adapt to real-world variations, thereby boosting its robustness and domain generalization. Subsequently, the model’s accuracy improved by 3% and 12% on two motor imagery datasets [32].

Fine-tuning was used in our study to improve the versatility of our model. Fine-tuning is one of the domain adaptation techniques which can help the model better adapt to the features and distribution of new data and improve the performance of the model in new environments [33]. In one landscape study, a deep learning model was pre-trained on the ImageNet dataset, being fine-tuned and applied to different medical imaging data. The pre-trained model was successfully applied to retinal optical coherence tomography and pneumonia diagnosis [34]. In our previous studies, we also successfully demonstrated the capability of fine-tuning in improving model performance on external cohort [10, 18].

We did not have information on potential confounding various such as BMI, alcohol intake, and dietary habits for our study participants. Although BMI is less frequently reported to affect the results of eNose breathprints, it can be associated with other diseases, such as diabetes, that may lead to distinct breathprints [35]. Dietary habits have previously been reported to influence VOC metabolites [36]. Lifestyle has also been noted to affect fecal VOCs [37]. On the other hand, one study investigating the impact of food intake on eNose breathprints suggested that the impact would be significant if the food intake occurred very recently, and two hours might be sufficient to avoid food-induced alterations in eNose breathprints [38]. In our study, we requested that participants fast for four hours prior to testing. However, the impact of the aforementioned factors may still warrant special attention and could be evaluated in future studies.

Our study has limitations. Firstly, the majority of lung cancer patients we studied in the study were in advanced stages, limiting the validation of eNose performance in early-stage lung cancer. Though the case number is limited, we have corrected identified two early stage lung cancer in our test cohort. Another limitation concerns transfer learning, which still necessitates some samples from the test cohort, potentially leading to inconvenience. While using data augmentation without fine-tuning yielded satisfactory results, fine-tuning can be viewed as a means to further optimize these results. Also, the study was confined to a Taiwanese population and the generalizability of the findings to other ethnicities remains uncertain. Finally, the reduced performance of eNose among elderly individuals and smokers also necessitates further investigation and strategies for improvement.

## Conclusion

In conclusion, our study has shown that cross-site validation of the electronic nose for diagnosing lung cancer is attainable. Data augmentation and fine-tuning have demonstrated to be crucial methods for improving the performance when applying the eNose across different sites. Consequently, the electronic nose holds promise as a valuable tool for accurately identifying lung cancer patients in clinical practice. Future researches were warranted to further assess the generalization of eNose, minimize influence of confounding factors and validate eNose in early-stage lung cancer, diverse populations as well as high-risk groups.

### Electronic supplementary material

Below is the link to the electronic supplementary material.

Supplementary Material 1

Supplementary Material 2

Supplementary Material 3

Supplementary Material 4

Supplementary Material 5

Supplementary Material 6

## Data Availability

All data will be available upon reasonable request. Part of this study has been presented in IEEE BioSensors 2023 conference.

## Abbreviations

eNose: Electronic Nose
SDA: Semi-supervised Domain-generalized Augmentation
NSA: Noise-Shift Augmentation
AUC: Area Under the Curve
AUC-ROC: Area Under the Receiver Operating Characteristic
CT: Computed Tomography
CNN: Convoluted Neural Network
NTUH: National Taiwan University Hospital
NTUH-HC: National Taiwan University Hospital Hsin-Chu branch
COPD: Chronic Obstructive Pulmonary Disease
DM: Diabetes Mellitus
ESRD: End-Stage Renal Disease
MOS: Metal-Oxide-Semiconductor
ReLUs: Rectified Linear Units
CI: Confidence Intervals
PCA: Principal Component Analysis
SCLC: Small Cell Lung Cancer
SqCC: Squamous Cell Carcinoma
VOCs: Volatile Organic Compounds
SIFT-MS: Selected Ion Flow Tube Mass Spectrometry
