# Supplementary figures and images for: Cross-site validation of lung cancer diagnosis by electronic nose with deep learning: a multicenter prospective study
Source: Respir Res. 2024 May 10;25:203. doi: 10.1186/s12931-024-02840-z (PMC11084132; doi:10.1186/s12931-024-02840-z)

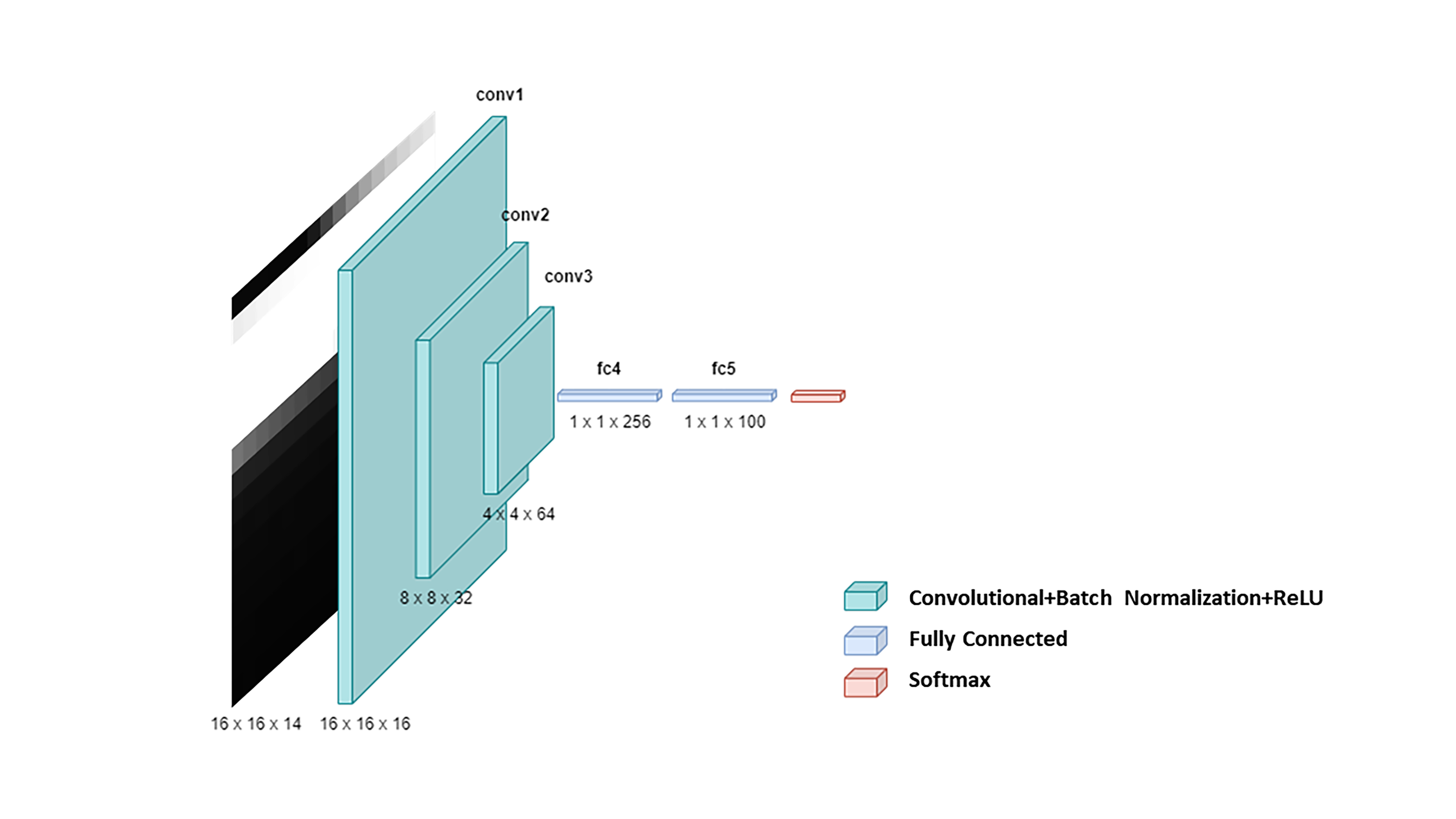

Supplement: Supplementary file 2 — Supplementary Material 2 [file 12931_2024_2840_MOESM2_ESM.tif]

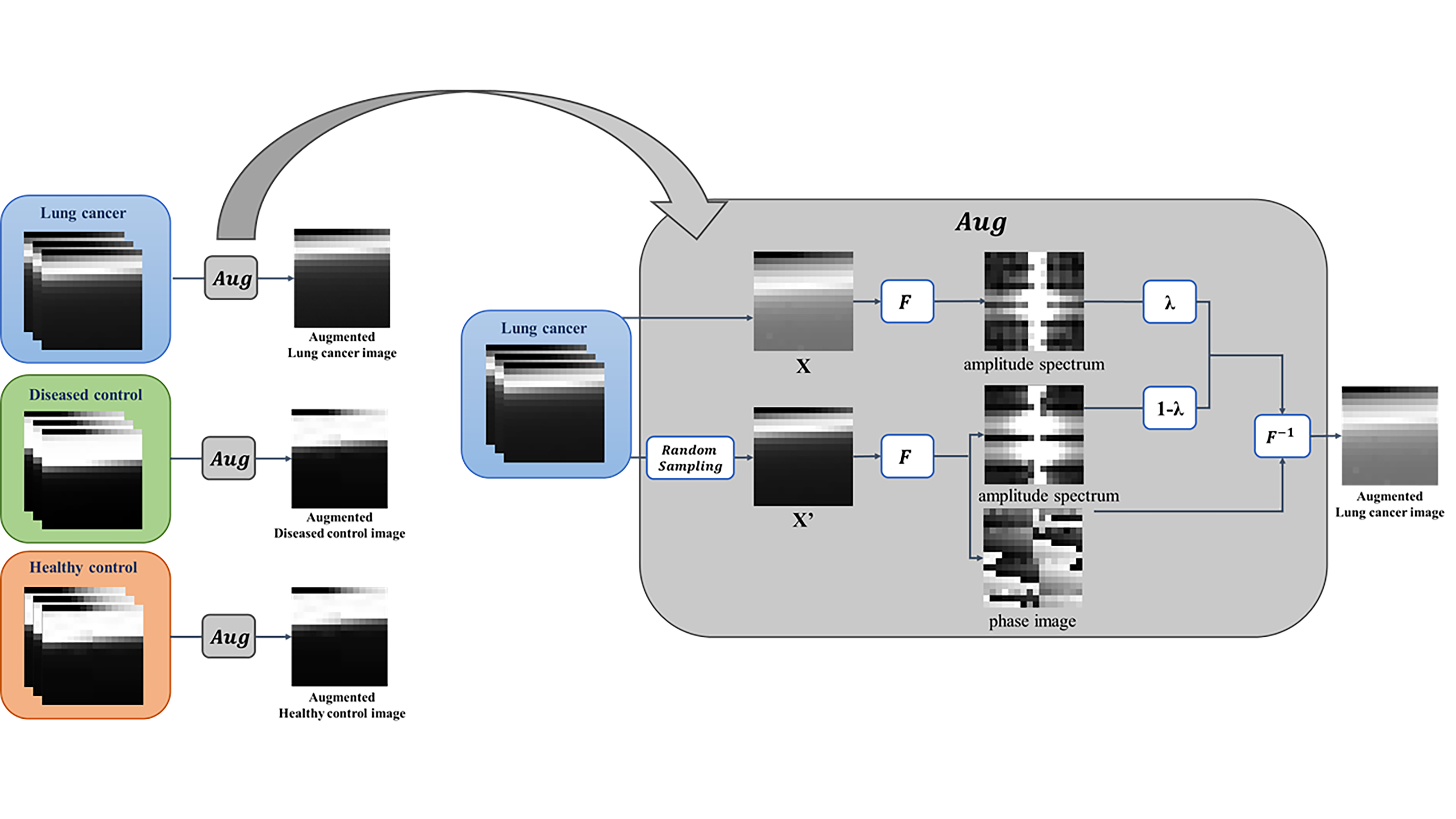

Supplement: Supplementary file 4 — Supplementary Material 4 [file 12931_2024_2840_MOESM4_ESM.tif]

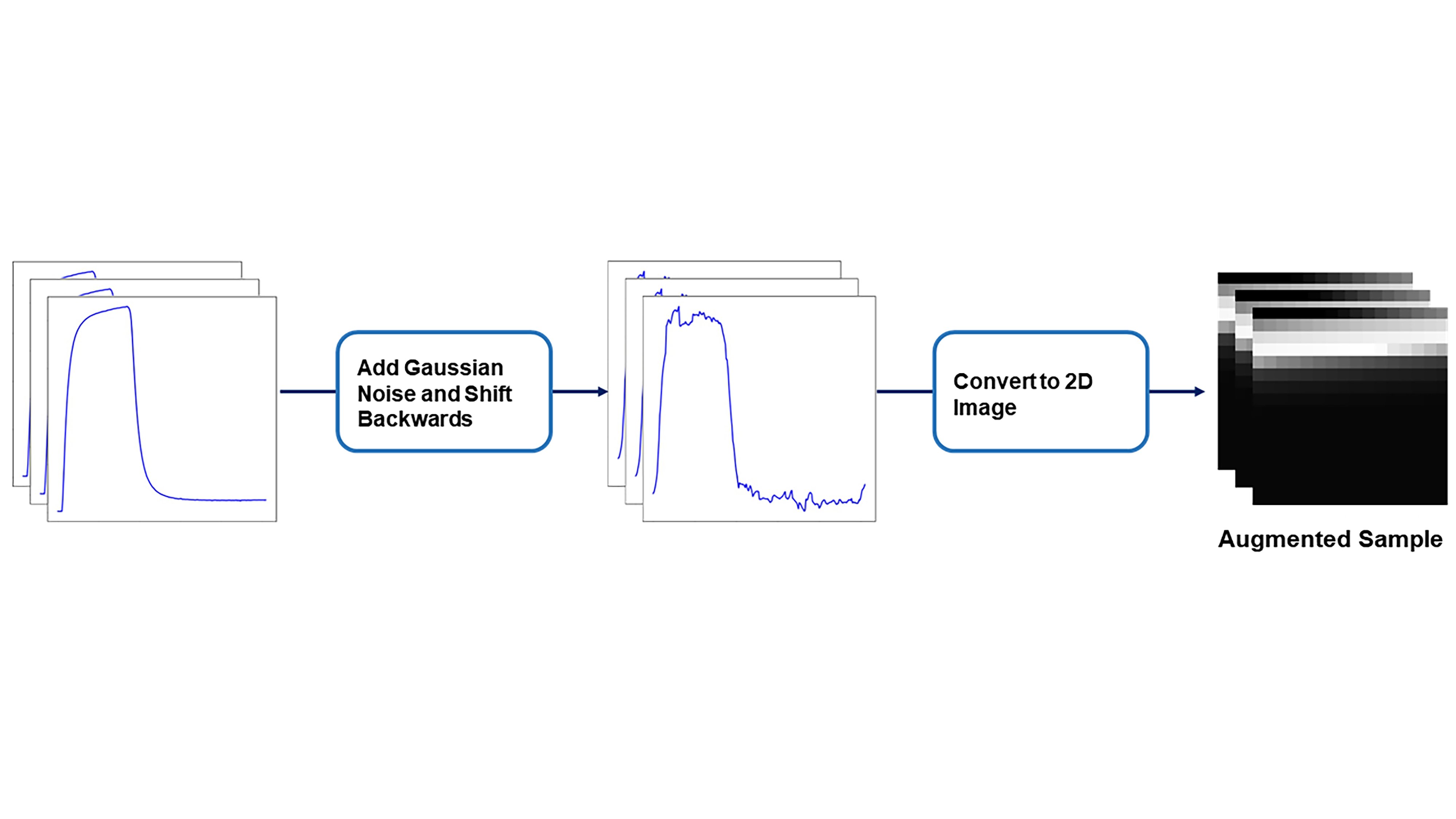

Supplement: Supplementary file 5 — Supplementary Material 5 [file 12931_2024_2840_MOESM5_ESM.tif]

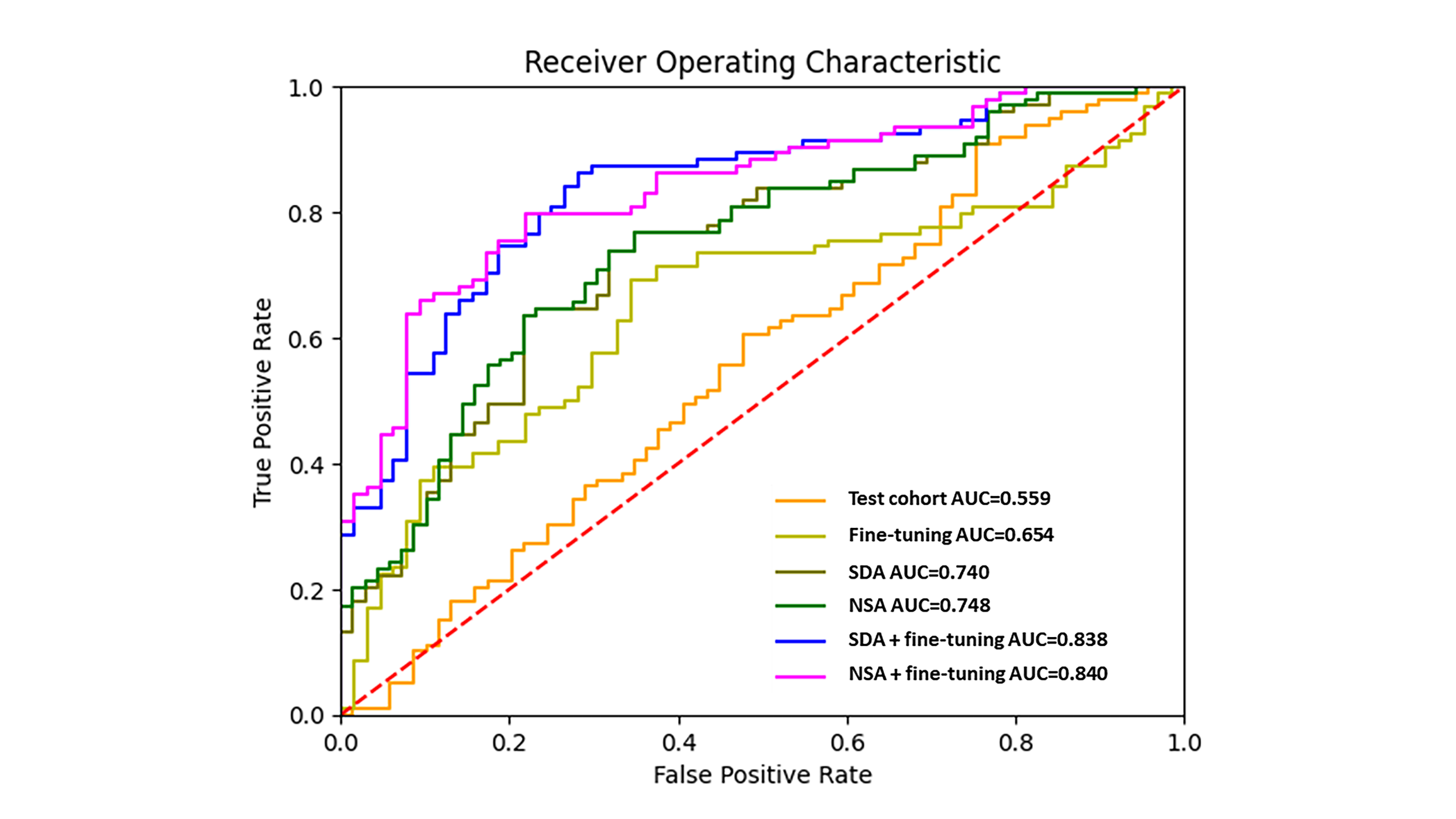

Supplement: Supplementary file 6 — Supplementary Material 6 [file 12931_2024_2840_MOESM6_ESM.tif]
