# Supplementary material for: Cross-site validation of lung cancer diagnosis by electronic nose with deep learning: a multicenter prospective study
Source: Respir Res. 2024 May 10;25:203. doi: 10.1186/s12931-024-02840-z (PMC11084132; doi:10.1186/s12931-024-02840-z)
Supplement: Supplementary file 3 — Supplementary Material 3 [file 12931_2024_2840_MOESM3_ESM.docx]

**Semi-DG (Semi-supervised domain generalized) data augmentation**

To reduce the impact of domain gap, we refer to the cross-domain information merging method as previously proposed with adjustment [1]. First, our data were divided into three categories: lung cancer, diseased control and healthy control. We adopted the strategy of converting and merging images of the same category. For example, images from the lung cancer group can only be merged with images from lung cancer group, vice versa.

Specifically, after conducting a Fourier transformation, we obtained a representation of the image in the frequency domain. The signal was expressed as the sum of sine and cosine functions of different frequencies. Amplitude represents the intensity of the signal in different frequency components while phase represents the starting position or offset of sine and cosine waves. In the Fourier domain, amplitude and phase are fundamental characteristics, providing crucial insights into the signal's structure and properties. For example, high-amplitude areas in an image may represent strong textures or edges while phase information can help us understand the arrangement and structure of different areas in the image. By merging the phase spectrum and amplitude spectrum of different images of the same category, we can preserve the spatial structure and texture features of the first image while incorporating the amplitude details of the second image. It can help maintain the features of the original image and add details of other images to enhance the image and improve domain generalization capabilities.

Therefore, our data augmentation process proceeded as follows: We began by selecting an image, X, for conversion and applied Fourier transformation (as per Formula 1) to transition it from the spatial domain to the frequency domain. We then converted it into amplitude spectrum and phase spectrum according to Formula 2 and Formula 3 respectively [2]. Next, we randomly chose an image, X', from the same category as X, and subjected it to the same aforementioned process to extract its amplitude and phase. Then, we merged the amplitude obtained from image X and the amplitude obtained from image X', and combined it with the phase obtained from the X’ to perform inverse Fourier transformation to generate an enhanced image. Our data augmentation method is further illustrated in **Additional File 4:** **Figure S2**.

**Formula 1, 2 and 3**

| $\boldsymbol{F}\left( \boldsymbol{u}, \boldsymbol{v} \right)=\sum_{\boldsymbol{x}=\mathbf{0}}^{\boldsymbol{M}-\mathbf{1}} \sum_{\boldsymbol{y}=\mathbf{0}}^{\boldsymbol{N}-\mathbf{1}} \boldsymbol{f}\left( \boldsymbol{x},\boldsymbol{y} \right)\times\boldsymbol{e}^{-\boldsymbol{j}\mathbf{2}\boldsymbol{\pi}(\frac{\boldsymbol{ux}}{\boldsymbol{M}}+\frac{\boldsymbol{vy}}{\boldsymbol{N}})}$ | Formula 1 |
| --- | --- |

, where $\boldsymbol{u}$ and $\boldsymbol{v}$ are coordinates in the frequency domain, $\boldsymbol{f}\left( \boldsymbol{x},\boldsymbol{y} \right)$ is the gray value of the image, $\left( \boldsymbol{x},\boldsymbol{y} \right)$ is the spatial coordinate of the image, $\boldsymbol{M}$ and $\boldsymbol{N}$ are the width and height of the image respectively.

| $\boldsymbol{A}\left( \boldsymbol{u}, \boldsymbol{v} \right)=\sqrt{{\boldsymbol{Re}(\boldsymbol{F}(\boldsymbol{u},\boldsymbol{v}))}^{\mathbf{2}}+{\boldsymbol{Im}(\boldsymbol{F}(\boldsymbol{u},\boldsymbol{v}))}^{\mathbf{2}}}$ | Formula 2 |
| --- | --- |

, where $\boldsymbol{Re}(\boldsymbol{F}(\boldsymbol{u},\boldsymbol{v}))$ and $\boldsymbol{Im}(\boldsymbol{F}(\boldsymbol{u},\boldsymbol{v}))$ are the real part and imaginary part of $\boldsymbol{F}(\boldsymbol{u},\boldsymbol{v})$ respectively.

| $\boldsymbol{\phi}\left( \boldsymbol{u}, \boldsymbol{v} \right)=\boldsymbol{tan}^{-\mathbf{1}}(\frac{\boldsymbol{Im}(\boldsymbol{F}(\boldsymbol{u},\boldsymbol{v}))}{\boldsymbol{Re}(\boldsymbol{F}(\boldsymbol{u},\boldsymbol{v}))})$ | Formula 3 |
| --- | --- |

**Noise-shift augmentation (NSA) methods**

To enable the collected signals to effectively simulate the changes and noise in the actual environment, we used traditional signal processing methods to augment the data [3]. We adopt a similar operation as previously described [4]. We first added Gaussian Noise to the collected eNose signal and then performed a backward shift operation. The Gaussian noises are generated from the standard derivation of the original training samples, with a normal distribution and different scales as defined in **Formula** **1**. Finally, we converted the processed signal into a 2D image. The detailed process is also illustrated in **Additional File 5:** **Figure S3.**

**Formula 1**

| $\boldsymbol{Noise}_{\boldsymbol{i}}\boldsymbol{=c\times}\boldsymbol{SD}_{\boldsymbol{i}}\boldsymbol{\times\delta}$***,*** $\boldsymbol{\delta\sim N(0,1)}$ | Formula 1 |
| --- | --- |

, where $\boldsymbol{i}$ denotes the i-th feature, $\boldsymbol{c}$ is the coefficient of noise level taking a value between (0.001, 0.015), $\boldsymbol{SD}_{\boldsymbol{i}}$ is the standard deviation, and $\boldsymbol{\delta}$ is a random number drawn from a normal distribution with mean 0 and standard deviation 1.

Reference

1. Yao H HX, and Li X. Enhancing pseudo label quality for semi-supervised domain-generalized medical image segmentation. *In Proceedings of the AAAI Conference on Artificial Intelligence* 2022: 36(3): 3099-3107.

2. N BR. The Fourier transform and its applications. McGraw-Hill, New York, 1978.

3. Stéphane M. A Wavelet Tour of Signal Processing The Sparse Way. 3rd ed. Elsevier, 2009.

4. Li Liu XZ, Rumeng Wu, Xiaoqing Guan, Zhan Wang, Wei Zhang, Mert Pilanci, You Wang, Zhiyuan Luo, Guang Li. Boost AI Power: Data Augmentation Strategies With Unlabeled Data and Conformal Prediction, a Case in Alternative Herbal Medicine Discrimination With Electronic Nose. *IEEE Sensors Journal* 2021: 21: 22995-23005.

**Table S1.** Lung cancer histologic classifications and stage in the training/validation cohort and test cohort

|  | **All** | **Training/Validation Cohort (S1, site 1, Hsin-Chu cohort)** | **Test Cohort (S2, site 2, Taipei cohort)** | **P value** |
| --- | --- | --- | --- | --- |
|  | **All (n=118)** | **Lung cancer (n=90)** | **Lung cancer (n=28)** |  |
| **Histology** |  |  |  |  |
| Adenocarcinoma | 85 (72.0) | 70 (77.8) | 15 (53.6) | 0.0165 |
| Squamous cell | 16 (13.6) | 12 (13.3) | 4 (14.3) |  |
| Small cell | 8 (6.8) | 4 (4.4) | 4 (14.3) |  |
| Other | 9 (7.6) | 4 (4.4) | 5 (17.9) |  |
| **Staging** |  |  |  | 0.5444 |
| I | 6 (5.1) | 5 (5.6) | 1 (3.6) |  |
| II | 2 (1.7) | 1 (1.1) | 1 (3.6) |  |
| III | 31 (26.2) | 22 (2.4) | 9 (32.1) |  |
| IV | 79 (66.9) | 62 (68.9) | 17 (60.7) |  |

**Table S2.** Diagnostic performance of eNose in the validation (S2) and test cohort (S1) after reversal

|  | AUC | 95% CI | Sensitivity | 95% CI | Specificity | 95%　CI | Accuracy | 95% CI |
| --- | --- | --- | --- | --- | --- | --- | --- | --- |
| Validation cohort (S2) | 0.91 | 0.81─1.00 | 0.89 | 0.80─1.00 | 0.80 | 0.60─1.00 | 0.84 | 0.60─1.00 |
| Test cohort (S1) | 0.56 | 0.44─0.73 | 0.63 | 0.52─0.76 | 0.54 | 0.48─0.60 | 0.59 | 0.46─0.72 |
| Test cohort (S1) with fine-tuning (n=10) | 0.65 | 0.54─0.76 | 0.72 | 0.68─0.80 | 0.66 | 0.59─0.73 | 0.69 | 0.61─0.83 |
| Test cohort (S1) with Semi-DG data augmentation in training cohort (S2) | 0.74 | 0.66─0.82 | 0.77 | 0.65─0.89 | 0.71 | 0.63─0.81 | 0.74 | 0.64─0.84 |
| Test cohort (S1) with NSA in training cohort (S2) | 0.75 | 0.68─0.82 | 0.77 | 0.65─0.89 | 0.71 | 0.63─0.81 | 0.74 | 0.64─0.84 |
| Test cohort (S1) with Semi-DG data augmentation in training cohort (S2) and fine-tuning (n=10) in test cohort (S1) | 0.84 | 0.78─0.90 | 0.82 | 0.73─0.90 | 0.79 | 0.70─0.89 | 0.81 | 0.72─0.90 |
| Test cohort (S1) with NSA in training cohort (S2) and fine-tuning (n=10) in test cohort (S1) | 0.84 | 0.78─0.90 | 0.82 | 0.73─0.90 | 0.79 | 0.70─0.89 | 0.81 | 0.72─0.90 |

NSA, noise-shift augmentation; SDA, semi-supervised domain generalized augmentation

**Table S3.** Detailed subgroup analysis among age, smoking status and comorbidities in the test cohort (S2)

|  | Sensitivity | 95% CI | Specificity | 95%　CI | Accuracy | 95% CI |
| --- | --- | --- | --- | --- | --- | --- |
| Age (By quartile) |  |  |  |  |  |  |
| <48 | 0.50 | 0─1.00 | 0.90 | 0.70─1.00 | 0.83 | 0.67─1.00 |
| 48-62 | 0.89 | 0.67─1.00 | 1.00 | 1.00─1.00 | 0.93 | 0.79─1.00 |
| 62-70 | 1.00 | 1.00─1.00 | 0.67 | 0.33─1.00 | 0.83 | 0.67─1.00 |
| ≥70 | 1.00 | 0.50─1.00 | 0.56 | 0.22─0.89 | 0.73 | 0.53─1.00 |
| Active smoker | 0.83 | 0.50─1.00 | 0.80 | 0.40─1.00 | 0.82 | 0.55─1.00 |
| Ever Smoker | 1.00 | 1.00─1.00 | 0.40 | 0.00─1.00 | 0.73 | 0.55─1.00 |
| Hypertension | 0.75 | 0.25─1.00 | 0.75 | 0.25─1.00 | 0.75 | 0.38─1.00 |
| DM | 1.00 | 1.00─1.00 | 0.75 | 0.25─1.00 | 0.89 | 0.67─1.00 |
| COPD | 1.00 | 1.00─1.00 | 0.71 | 0.43─1.00 | 0.83 | 0.67─1.00 |

COPD, chronic obstructive pulmonary disease; DM, diabetes mellitus
